# Eluxadoline Loaded Solid Lipid Nanoparticles for Improved Colon Targeting in Rat Model of Ulcerative Colitis

**DOI:** 10.3390/ph13090255

**Published:** 2020-09-19

**Authors:** Md. Khalid Anwer, Mohammed Muqtader Ahmed, Mohammed F. Aldawsari, Saad Alshahrani, Farhat Fatima, Mohd Nazam Ansari, Najeeb Ur Rehman, Ramadan I. Al-Shdefat

**Affiliations:** 1 Department of Pharmaceutics, College of Pharmacy, Prince Sattam Bin Abdulaziz University, Alkharj 11942, Saudi Arabia; mo.ahmed@psau.edu.sa (M.M.A.); moh.aldawsari@psau.edu.sa (M.F.A.); sm.alshahrani@psau.edu.sa (S.A.); f.soherwardi@psau.edu.sa (F.F.); 2 Department of Pharmacology and Toxicology, College of Pharmacy, Prince Sattam Bin Abdulaziz University, Alkharj 11942, Saudi Arabia; m.ansari@psau.edu.sa (M.N.A.); n.rehman@psau.edu.sa (N.U.R.); 3 Department of Pharmaceutical Sciences, Faculty of Pharmacy, Jadara University, Irbid 21110, Jordan; rshdefat@yahoo.com

**Keywords:** eluxadoline, lipid, solid lipid nanoparticles, in vitro release, ulcerative colitis

## Abstract

The aim of the current study was to evaluate the therapeutics potential of eluxadoline (ELX) loaded solid lipid nanoparticles (SLNs) in ulcerative colitis. ELX loaded SLNs were prepared using three different lipids according to the solvent emulsification technique. The optimization of prepared SLNs (F1-F3) were carried out based on size, PDI, zeta potential, percent drug entrapment (%EE), and loading (%DL). The lipid (stearic acid) based SLNs (F2) was optimized with particle size (266.0 ± 6.4 nm), PDI (0.217 ± 0.04), zeta potential (31.2 ± 5.19 mV), EE (65.0 ± 4.8%), and DL (4.60 ± 0.8%). The optimized SLNs (F2) was further evaluated by DSC, FTIR, SEM, in vitro release, and stability studies, which confirmed the successful encapsulation of ELX in SLNs. The efficacy of optimized SLNs (F2) in comparison to the pure ELX drug was assessed in acetic acid induced colitis rat models. It was observed that the delivery of ELX by SLNs alleviated the induced acetic acid colitis significantly. Thus, ELX loaded SLNs delivery to the colon has a significant potential to be developed for the treatment of ulcerative colitis.

## 1. Introduction

Irritable Bowel Syndrome (IBS) is a widespread and chronic disorder that affects the colon’s motility and ability to work properly [1]. Abdominal pain, cramps, gas, bloating, diarrhea, and/or constipation are common signs and symptoms of IBS. In many cases, the exact cause of illness is unknown, so the goal of treatment is to improve the symptoms of the IBS [2,3]. Eluxadoline (ELX) is the choice of drug for IBS but it can be useful for the amelioration of pain and gastrointestinal disorders such as ulcerative colitis and Crohn’s disease [4]. Ulcerative colitis (UC) is a critical disease condition of ulcer and inflammation of the inner lining of the colon that causes rectal bleeding, diarrhea, abdominal pain, nausea, vomiting, anorexia, and fever [5]. UC can occur in people between 15 to 30 years of age and in both sexes.

ELX is a new oral drug indicated in adults for the treatment of irritable bowel syndrome with predominant diarrhea (IBS-D). It acts uniquely and locally on three different opioid receptors (μ-and κ-opioid receptor agonists and δ-receptor antagonist), significantly improving IBS-D symptoms, bowel function, and patients’ quality of life [6,7]. However, ELX exhibits low oral bioavailability and high level of inter-subject variability in patients due to its poor water solubility, significant presystemic metabolism, and low gastrointestinal permeability. Furthermore, the median area under the plasma concentration-time profile (AUC) and the maximum concentration of ELX in plasma (Cmax) were reported from 12 to 22 ng·h/mL and 2 to 4 ng/mL, respectively, after the oral administration of 100 mg tablets daily in healthy subjects [8,9,10]. To address these problems, numerous promising strategies of ELUX are necessary in order to achieve efficient, targeted drug delivery and improve the ELUX oral bioavailability by enhancing its solubility, dissolution rate, and permeability as carrier complexes, nanocrystals, polymeric nanoparticles, dendrimers, or other drug delivery systems [11,12,13].

Next generation lipid-based nanoparticle drug delivery technologies, such as solid lipid nanoparticles (SLNs) and nanostructured lipid carriers, have shown to be a promising alternative to other colloidal carriers, such as liposomes and microemulsions [14,15]. SLNs are composed of lipid matrices that remain in a solid state at both room and body temperatures. These particles are in the size range of 50–1000 nm [16]. SLNs offer exceptional advantages, having low toxicity, improving the bioavailability and stability of the drug, as well as the possibility to incorporate hydrophilic or lipophilic drugs into SLNs [17,18]. Among the various nano-carriers, SLNs are promising nanoparticle drug delivery carriers for the treatment of ulcerative colitis. SLNs have been proposed as an alternative colloidal carrier for drug delivery due to its various advantages, including the feasibility of the incorporation of lipophilic and hydrophilic drugs, drug targeting, improved stability, low cost, and ease of manufacturing. SLNs are composed of solid lipids, an emulsifier and water/solvent [19].

The purpose of this study was to develop and optimize a successful oral colon targeted ELX loaded SLNs for the treatment of ulcerative colitis. We have used three different lipids, stearic acid, glyceryl monostearate, and glyceryl trioctoanate, for the preparation of SLNs. The effects of lipids on the particle size, zeta potential, and drug encapsulation were studied in order to optimize a suitable lipid for the preparation of SLNs. The ELX loaded SLNs were developed in order to increase the aqueous solubility and prevent acidic and enzymatic degradation that would eventually increase the bioavailability and hence improve patient compliance, leading to a better control of ulcerative colitis.

## 2. Results and Discussion

### 2.1. Particles Characterization

Table 1 presents the mean particle size, PDI, and zeta potential of all three prepared SLNs (F1–F3). The particle sizes for all SLNs were found to be in the range of 266.0 ± 6.4 to 1570.5 ± 14.2 nm. PDI is used to identify the degree of non-uniformity of particle size distribution. A PDI below 0.3 indicates the uniform distribution of nanoparticles [20]. For the developed SLNs (F1–F3), the PDI was observed in the range of 0.226 ± 0.03 to 0.882 ± 0.06. The measurement of zeta potential is another particle characterization parameter which is directly correlated with the stability of colloidal particles. The zeta potential of SLNs (F1–F3) were measured in the range of 25.7 ± 6.11 to 31.2 ± 5.19 mV. The molecular weight of stearic acid, glyceryl monostearate, and glyceryl trioctonoate used in SLNs preparation were 248.48 g/mol, 358.57 g/mol, and 470.7 g/mol, respectively. The molecular weight of lipids directly influences the size and zeta potential. The results showed that an increase in size and zeta potential occurs with increase in molecular weight [21]. Based on particles size, PDI, and zeta potential, the SLNs with lipid composition of stearic acid (F2) was found to be best among all the SLNs.

### 2.2. Percent Drug Entrapment and Loading

**Figure 1:**
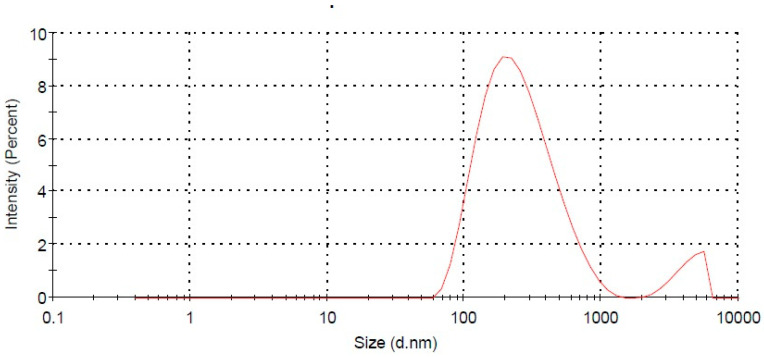
Particle size of optimized SLNs (F2) measured by the dynamic light scattering (DLS) method.

The lipid core materials were found to affect the extent of ELX entrapment in the prepared SLNs. The %EE and %DL of the prepared ELX loaded SLNs (F1–F3) were measured in the range of 54.1 ± 2.6% to 73.0 ± 3.2% and 3.82 ± 0.7% to 5.13 ± 1.2%, respectively (Table 1). The SLNs (F1 and F2) showed a considerably high entrapment of the drug. Among the three prepared SLNs, the ELX loaded SLNs (F2) containing stearic acid was found to be optimum in terms of size (266.0 ± 6.4 nm), PDI (0.217 ± 0.04), zeta potential (31.2 ± 5.19 mV), EE (65.0 ± 4.8%), and DL (4.60 ± 0.8%) (Figure 1). The optimized EXL loaded SLNs (F2) was further studied for spectral analysis, in vitro release studies, and ulcerative colitis assessment in an animal model.

### 2.3. Thermal Analysis

**Figure 2:**
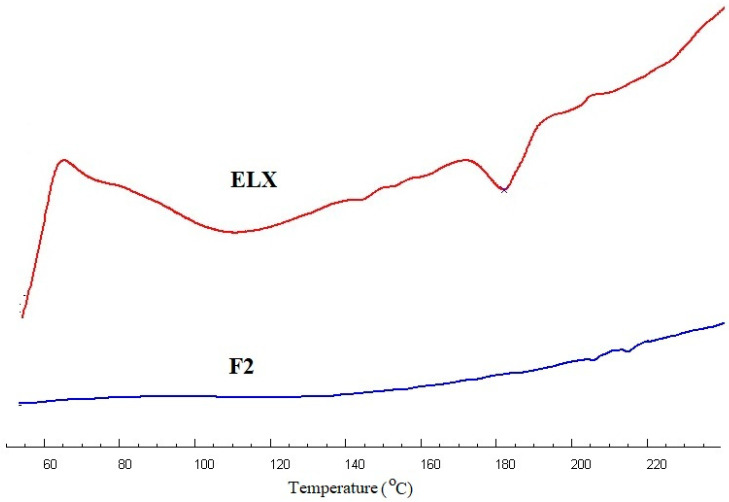
DSC spectra of pure ELX and ELX loaded SLNs (F2).

The thermal behavior of the pure ELX and optimized SLN (F2) in the temperature range of 50–240 °C is shown in the thermographs in Figure 2. The DSC thermogram of pure ELX showed a broad endothermic peak at 186 °C, which is close to the reported melting temperature of the drug [22]. The complete disappearance of the endothermic peak corresponding to the drug in optimized SLNs (F2) evidenced the encapsulation.

### 2.4. FTIR Spectra Analysis

**Figure 3:**
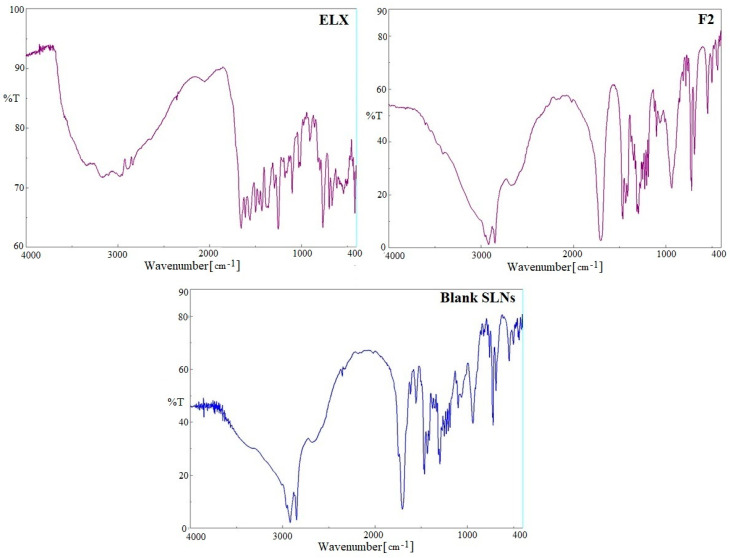
FTIR spectra of pure eluxadoline (ELX), blank SLNs and optimized ELX loaded SLNs (F2).

A comparative FTIR spectral analysis of pure ELX, blank SLNs and optimized ELX loaded SLNs (F2) were carried out for the identification of encapsulated drug inside SLNs (Figure 3). The prominent peaks of pure ELX were assigned corresponding to functional groups, aliphatic primary amines (3346 cm^−1^), acid carbonyl (1654 cm^−1^), amide carbonyl (1608 cm^−1^), –C=C-str (1429 cm^−1^), –O–H-str (3175 cm^−1^) and –C–H-str (2962 cm^−1^), confirmed the identification of drug. Various peaks in FTIR spectra of blank SLNs were observed due to presence of stearic acid and soyalecithin. These blank SLNs peaks were preserved in the ELX loaded SLNs (F2) without any shifts, which suggests encapsulation of ELX inside lipid matrix.

### 2.5. In-Vitro Drug Release Profile

**Figure 4:**
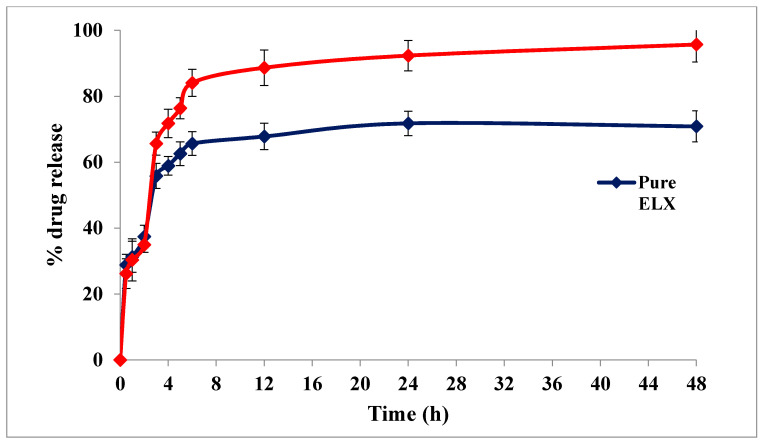
A comparative in vitro release profile of pure ELX and ELX loaded SLNs (F2).

The in-vitro drug release profiles of pure ELX and optimized ELX loaded SLNs (F2) were tested at progressive pH 1.2 and 7.4 for 48 h (Figure 4). The release of pure ELX was observed to be 37.42% after 2 h at pH 1.2, but ELX loaded SLNs (F2) showed 34.97% drug release, which was less compared to pure ELX. This is probably because the drug was protected from the acidic environment due to its entrapment inside the lipid core. However, when F2 moved into the intestine (pH 7.4), the release of ELX reached its peak level (95.71%) in comparison to pure ELX (70.86%) after 48 h. It was concluded from the release results that SLNs showed a slow release at acidic pH which reached a maximum at pH 7.4 with a sustained pattern of release [23].

### 2.6. Morphology

**Figure 5:**
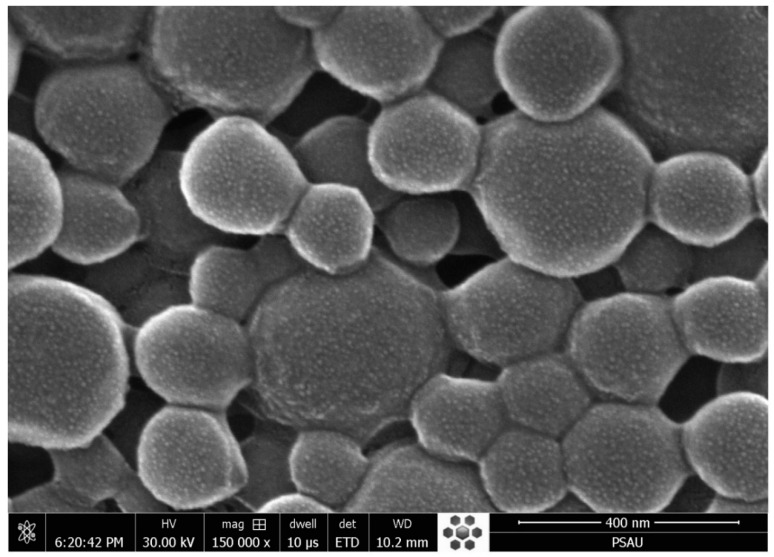
SEM images of optimized ELX loaded SLNs (F2).

The SEM image of optimized SLNs (F2) is shown in Figure 5. It was observed that the SLNs were spherical in shape, with a smooth surface. It was noticed that particles adhered together, probably due to nature of the soyalecithin used in the formulation.

### 2.7. Stability Studies

The stability of optimized F2 was assessed after three months in terms of particle size, PDI, ZP, and entrapment efficiency after storage as per ICH guidelines. After three months of storage at two conditions, 30 ± 2 °C/65 ± 5% RH and 40 ± 2 °C/75 ± 5% RH in stability chamber, no\significant change was observed in particle size, PDI, ZP, and entrapment efficiency (Table 2). The stability data revealed that the optimized F2 was physically stable.

### 2.8. In Vivo Studies: Assessment of Ulcerative Colitis

#### 2.8.1. Effect of ELX and F2 on Disease Activity Index (DAI)

**Figure 6:**
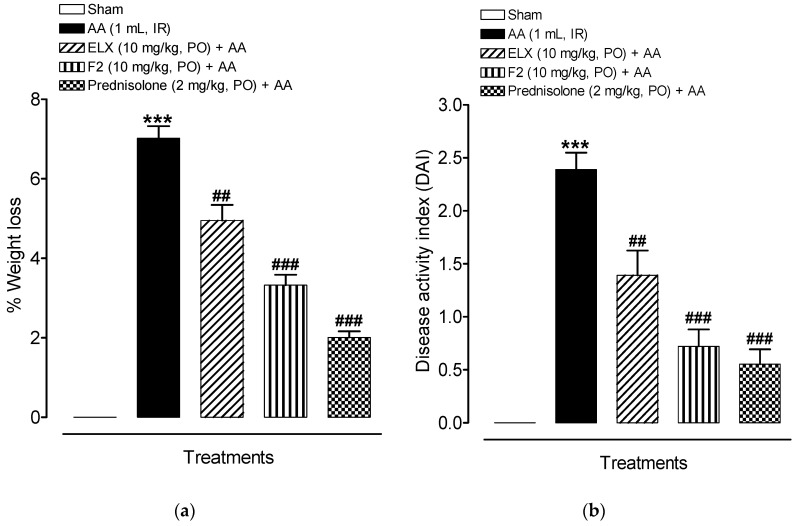
Pretreatment with ELX and F2 improved percentage of (**a**) weight loss and (**b**) disease activity index (DAI) in Wistar albino rats induced with AA colitis. Data are represented as means ± SEM (*n* = 6). *** *p* < 0.001 compared with sham group; ^##^ *p* < 0.01, ^###^ *p* < 0.001 compared with AA alone. ELX = Eluxadoline, F2 = formulation, AA = acetic acid; PO = orally; IR = intrarectally. Note: Normal stools = well-formed pellets, loose stools = pasty and semi formed stools which do not stick to the anus, and diarrhea = liquid stools that stick to the anus.

The disease was noted for 24 h after the IR administration of AA in the respective groups. The AA control animals group underwent distinct weight loss (*p* < 0.001) due to severe AA induced anorexia as shown in Figure 6a. However, the weight loss was significantly lower in the ELX (*p* < 0.01), F2 *p* < 0.001) and prednisolone (*p* < 0.001) pre-treated groups when compared with AA group.

The overall DAI were reduced in all treated groups, as can be seen in Figure 6b. The AA group showed a consistent increase in DAI (*p* < 0.001) whereas ELX (*p* < 0.01) and F2 (*p* < 0.001) reduced the DAI.

#### 2.8.2. Effect of ELX and F2 on Macroscopic Damage

**Figure 7:**
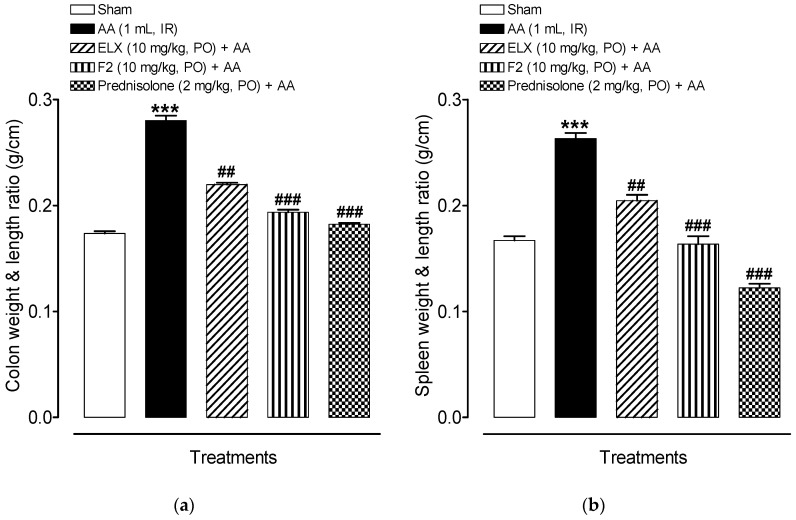
Pre-treatment with ELX and F2 decreased (**a**) colon wet weight and length ratio, and (**b**) spleen wet weight and length ratio in Wistar albino rats induced with AA colitis. Data are represented as means ± SEM (*n* = 6). *** *p* < 0.001 compared with sham group; ^##^ *p* < 0.01, ^###^ *p* < 0.001 compared with AA alone. ELX = Eluxadoline, F2 = formulation, AA = acetic acid; PO = orally; IR = intrarectally.

While evaluating the wet weight/length ratio of rat colon and spleen, it was noticed that the AA group had a higher average weight/length ratio of the colon and spleen in comparison to the sham control (*p* < 0.001) whereas ELX (*p* < 0.01), F2 (*p* < 0.001) and prednisolone (*p* < 0.001) treated groups had relatively smaller ratios as compared to AA group, indicating a possible reduction in the inflammatory process, as shown in Figure 7a,b.

**Figure 8:**
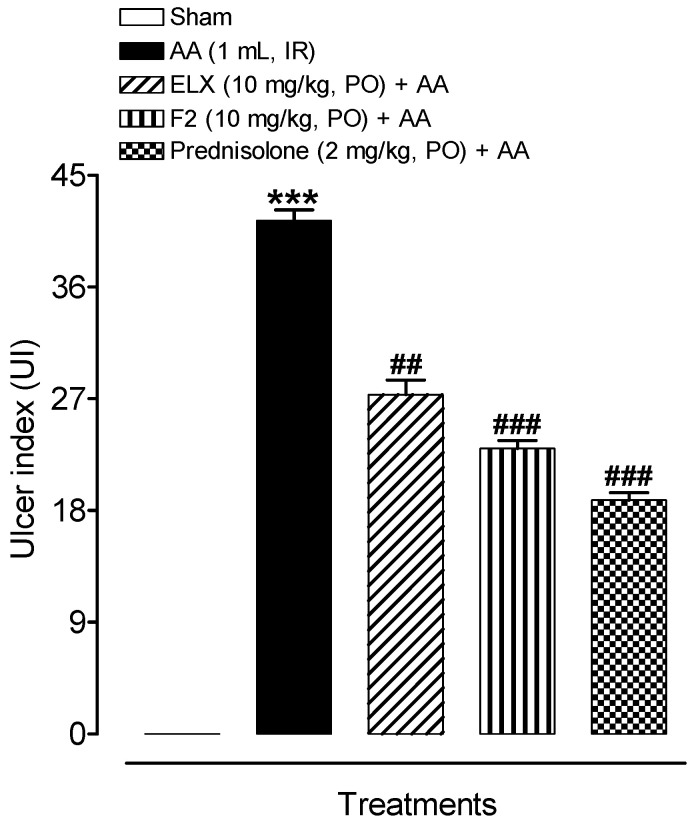
Pre-treatment with ELX and F2 decreased ulcer index in Wistar albino rats induced with AA colitis. Data are represented in means ± SEM (*n* = 6). One-way ANOVA was used for statistical analysis followed by Tukey’s post-test. *** *p* < 0.001 compared with sham group; ^##^ *p* < 0.01, ^###^ *p* < 0.001 compared with AA alone. ELX = Eluxadoline, F2 = formulation, AA = acetic acid; PO = orally; IR = intrarectally.

The AA treated rat colon showed a flaccid appearance and bowel wall thickening, and ulcers were also noticed. The ulcer lesion score and ulcer area were assessed for all the groups, and a decreasing trend was evident in the ELX, F2, and prednisolone treated groups. Figure 8 shows the sum of lesion score and ulcer area represented as ulcer index. The AA group had highest ulcer index (38.57 ± 2.86) and pretreatment with ELX, F2, and prednisolone significantly reduced this ulcer index to 26.57 ± 1.25, 23.28 ± 0.61, and 19.28 ± 0.68, respectively. Hence, ELX and F2 significantly attenuated the extent and severity of the colonic injury, as compared to the AA group.

#### 2.8.3. Effect of ELX and F2 on Lipid Peroxidation Activity

**Figure 9:**
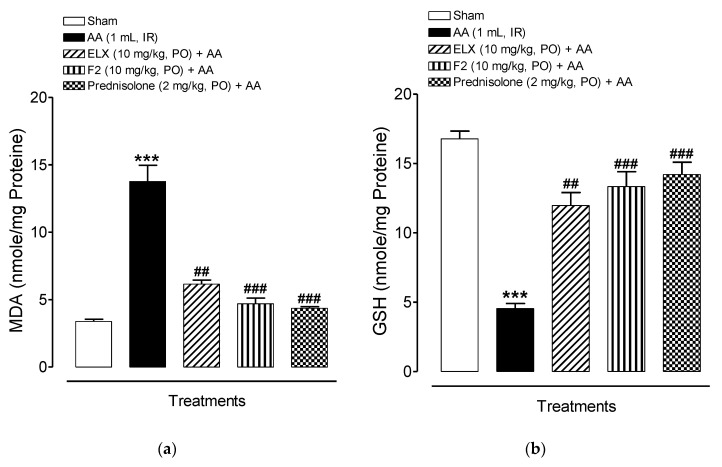

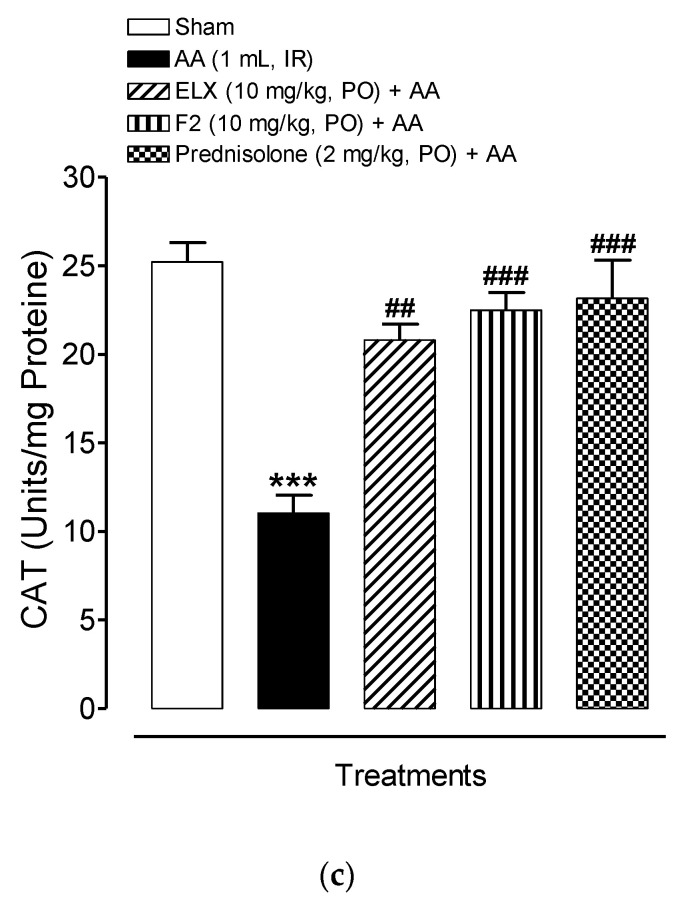
Pre-treatment with ELX and F2 improved (**a**) MDA, (**b**) GSH, and (**c**) CAT in Wistar albino rats induced with AA colitis. Data are represented in means ± SEM (*n* = 6). One-way ANOVA was used for statistical analysis followed by Tukey’s post-test. *** *p* < 0.001 compared with sham group; ^##^ *p* < 0.01, ^###^ *p* < 0.001 compared with AA alone. ELX = Eluxadoline, F2 = formulation, AA = acetic acid, PO = orally, IR = intrarectally, MDA = malondialdehyde, GSH = total glutathione, CAT = catalase.

Figure 9a shows the MDA activity that was measured as a marker of the lipid peroxidation (LPO) status in tissue homogenates of all the experimental rats. The AA group showed a significant (*p* < 0.001) increase in MDA (13.76 ± 1.21 nmol/mg protein) as compared to the sham control (3.39 ± 0.16 nmol/mg protein). Pre-treatment with ELX and F2 caused a significant reduction in MDA level as compared to the AA group. Prednisolone, the reference drug, also significantly reduced MDA levels. 

#### 2.8.4. Effect of ELX and F2 on Antioxidant Activity

The ulcerative colitis (AA) group exhibited a significant decrease in endogenous enzymatic (catalase) and non-enzymatic (total glutathione) antioxidants activity in the colonic tissue of rats, however, ELX, F2, and prednisolone pretreatments consistently elevated the colonic anti-oxidant activity compared to the AA group (Figure 9b,c).

Figure 9b shows that the total glutathione level was decreased in the AA group (4.54 ± 0.36 nmole/mg protein) as compared to sham control (16.78 ± 0.56 nmole/mg protein). ELX and F2 showed an increase in activity compared to the AA group. The positive control, prednisolone, also significantly increased total glutathione activity (14.21 ± 0.88 nmole/mg protein).

Figure 9c shows that CAT activity was reduced in AA group (11.03 ± 1.02 Units/mg proteins) as compared to the sham control (25.22 ± 1.08 Units/mg proteins). A significant increase in CAT activity was evident in the ELX and F2 treatment groups (20.80 ± 0.91 Units/mg proteins and 22.49 ± 1.01 Units/mg proteins, respectively). Prednisolone treatment also resulted in significantly increased CAT activity (23.17 ± 2.15 Units/mg proteins). 

Hence, ELX and F2 demonstrated a reliable increment in antioxidant activity in comparison to the AA group. In general, an inverse relation between antioxidant activity in the treatment group and the AA group was established, which may be the reason for attenuating disease severity in treated rats.

#### 2.8.5. Effect of ELX and F2 on Microscopic Damage

Pretreatment with ELX, F2, and prednisolone improved the histopathological changes in colon tissues in AA-induced colitis in Wistar albino rats (300×).

**Figure 10:**
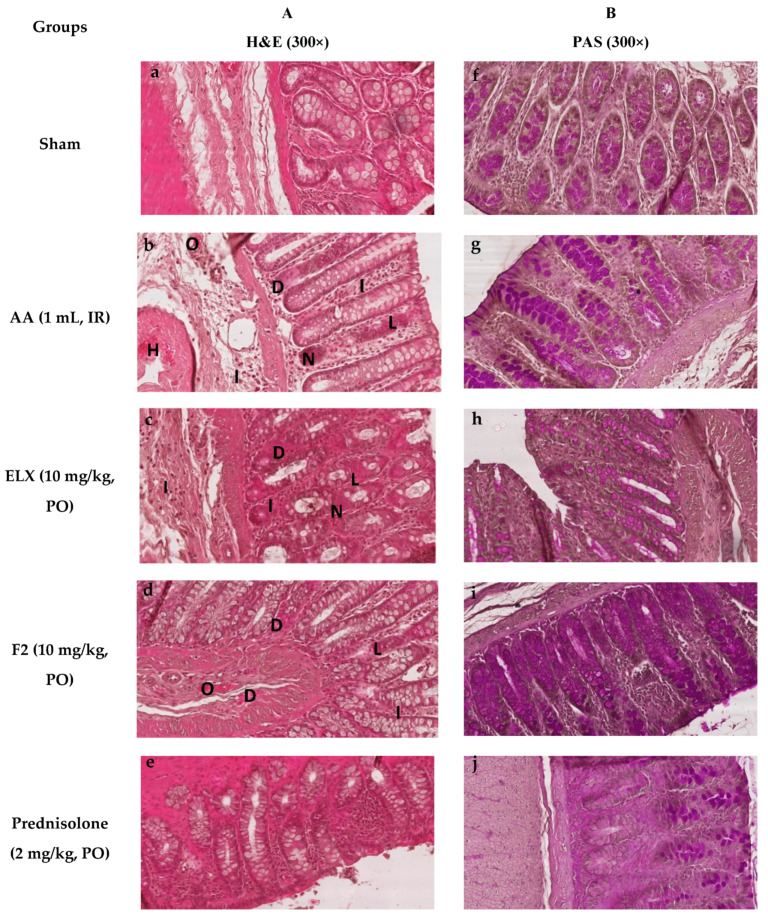
Impact of pure ELX and ELX loaded SLNs (F2) on histopathological changes in colon tissue in AA-induced colitis in Wistar albino rats (300×). Colon tissue were stained with (**A**) hematoxylin and eosin (H&E), and (**B**) PAS for microscopic evaluation. Sham control rats showing clear morphological structure. AA administered rats with H&E stain showed severe tissue damage of both mucosal and submucosal layers of intestine by showing areas of complete loss of goblet cells (L), remarkable necrosis (N), degeneration (D), as well as occlusion (O) of blood vessels and infiltration (I) of mucosa and submucosa by inflammatory cells. PAS staining showed severe loss of mucin and goblet cells. Pretreated rats with F2 showed better improvement compared to ELX whereas prednisolone showed complete protection against AA induced colon damage. ELX = Eluxadoline, F2 = formulation, AA = acetic acid, PO = orally, IR = intrarectally, H&E = hematoxylin and eosin, PAS = periodic acid Schiff’s.

Figure 10a–e shows the photographs of the tissue sections, which was processed for histological analysis and stained with hematoxylin and eosin (H&E). Sham treated rat tissues showed a normal morphological appearance (Figure 10a). AA treated rats displayed severe tissue damage of both the mucosal and submucosal layers of the intestine, showing areas of complete loss of goblet cells, remarkable necrosis, degeneration and occlusion of blood vessels and infiltration of mucosa and submucosa by inflammatory cells (Figure 10b). ELX, F2, and prednisolone treatment groups showed a marked decrease in ulcer, goblet cell depletion, and infiltration, which was mostly limited to the submucosal layer (Figure 10c–e).

Figure 10f–j represents the histograms, stained with PAS stain to assess goblet cells depletion as well as the type of mucin (neutral, acidic, basic). Figure 10f of the sham group shows the normal goblet cell that stained pink for neutral mucin at the mucosal layer. In the AA group, the goblet cells stained purple showing acidic mucin in the regions of the re-epithelized mucosal layer, whereas marked mucin depletion was evident in ulcerated areas (Figure 10b). Pretreatment with ELX, F2, and prednisolone showed relatively reduced depletion and retention of neutral mucin at the mucosal layer (Figure 10h–j).

Pretreated rats with F2 show better improvement compared to ELX, whereas prednisolone showed complete protection against AA induced colon damage.

## 3. Materials and Method

### 3.1. Materials

Eluxadoline was procured from Beijing Mesochem Technology Co., Ltd., Beijing, China. The lipids (Stearic acid, Glyceryl tristearate, and Glyceryl trioctanoate), prednisolone, and soyalecithin were procured from Sigma–Aldrich (St. Louis, MO, USA).

### 3.2. Preparation of ELX Loaded Solid Lipid Nanoparticle (SLNs)

The solid lipid nanoparticles (SLNs) were prepared by a solvent emulsification. Briefly, a specified amount (400 mg) of lipids (Stearic acid, Glyceryl tristearate, and Glyceryl trioctanoate) was dissolved in organic phase (dichloromethane) and 40 mg of ELX was added in solution (Table 3). The prepared lipid solution was emulsified by stirring to a 0.5% soyalecithin solutions (25 mL) and resultant dispersion was immediately sonicated for 3 min using a probe sonicator at an amplitude of 60% with the pulse of 10 s off-on intervals. After probe sonication the organic solvents present in the solution was evaporated on magnetic stirrer for 24 h at 100 rpm. The developed SLNs were lyophilized (Millirock Technology, Kingston, NY, USA) to yield amorphous powder.

### 3.3. Particles Characterization

The average particle size, PDI and zeta potential of prepared SLNs (F1-F3) were computed using a Malvern zetasizer (ZEN-3600, Malvern Instruments Ltd., Westborough, MA, USA) at 25 ± 2 °C [23]. The colloidal suspension of ELX loaded SLNs were diluted to 200 times with deionized water and ultrasonicated for 5 min, then the diluted sample (1.5 mL) was kept in the sample holder of the instrument in disposable plastic cuvette, after which the particle size and PDI were measured three times. 

The zeta potential of SLNs was measured following same procedure except using a glass electrode cuvette in the place of the glass cuvette. 

### 3.4. Percent Drug Entrapment (%EE) and Loading (%DL)

The %EE and %DL of prepared ELX loaded SLNs (F1–F3) were measured by separating the aqueous phase free drug by high speed cold centrifugation (15,000 rpm for 15 min). The clear aqueous supernatant was collected and content of free ELX was measured by following a previously referenced HPLC method after some modification [24]. The %EE and %DL were calculated by using following equation:

%EE = W_total ELX_ − W_free ELX_/W_total ELX_

%DL = W_total ELX_ − W_free ELX_/W_total SLNs_

### 3.5. Thermal Analysis

DSC thermal analysis of pure ELX and optimized SLNs (F2) were recorded using differential scanning calorimetry (Scinco, DSC N-650, Seoul, Korea). Each sample (approx. 5 mg) was weighed and crimped into an aluminum pan by applying pressure. The pressed pan was placed in the sample holder of DSC analyzer and heated from a temperature of 50° C to 240° C at a heating rate of 20 °C/min. The DSC analyzer was purged with inert N_2_ gas (20 mL/min) during the DSC procedure [23].

### 3.6. FTIR Spectra Analysis

FTIR spectral analysis of pure ELX, blank SLNs and optimized ELX loaded SLNs (F2) were recorded by using an FTIR spectrometer (Jasco FTIR-4700 Spectrophotometer, Tokyo, Japan) with a maximum resolution of 0.4 cm^−1^. Transparent pellets of samples were prepared by diluting with potassium bromide (1:10). The recorded FTIR spectra in the wavelength range of 400–4000 cm^−1^ were interpreted using spectra manager IR software.

### 3.7. Morphology 

The surface morphology of optimized SLNs (F2) was studied using SEM (JSM-6360LV Scanning Electron Microscope, Jeol, Tokyo, Japan). The suspension of SLNs (F2) was vortexed for 3 min, spread as a film on glass slide, and air dried and scanned for imaging [25].

### 3.8. In-Vitro Release Studies

Release studies of pure ELX and optimized ELX loaded SLNs (F2) were conducted in simulated gastric fluid (SGF, pH-1.2) and simulated intestinal fluid (SIF, pH-7.4); these pHs in the release studies were chosen due to the variation of pH in the gastrointestinal tract (GIT) in the stomach (pH ~ 1.5), and the colon (pH 7 to 7.8) [21]. Accurately weighed pure ELX and F2 SLNs were suspended in 25 mL of SGF media under and kept on biological shaker with stirring of 100 rpm for 2 h. The pH of this medium was changed to 7.4 by adding KH_2_PO_4_ (102 mg) and Na_2_HPO_4_.2H_2_O (155 mg) to achieve colon sink condition. The release study was continued for 48 h and drug was quantified by HPLC [26].

### 3.9. Stability Studies

The optimized F2 formulae were further subjected to stability study as per ICH guidelines in order to determine suitable storage conditions. Freeze dried powder of F2 was kept and sealed in amber colored vials and stored at 30 ± 2 °C/65 ± 5% RH and 40 ± 2 °C/75 ± 5% RH in stability chamber [27]. The SLNs was reconstituted with double distilled water for 5 min and evaluated for particle size, PDI, ZP, and entrapment efficiency after 0, 1, 2 and 3 months.

### 3.10. In Vivo Studies: Assessment of Ulcerative Colitis

#### 3.10.1. Experimental Animals

Wistar albino rats weighing 180–200 g were used in the ulcerative colitis study. The animals were procured from the Animal Care Unit, College of Pharmacy, Prince Sattam bin Abdulaziz University, Saudi Arabia, and housed under conditions of temperature (22 ± 1 °C), RH (55 ± 5%), and 12 h/12 h light/dark cycle. The animals were fed a standard diet with water ad libitum. ELX loaded SLNs (F2) and prednisolone were administered orally (p.o.). During the experimental period, the animals were maintained in accordance with the guidelines of the Institute of Laboratory Animal Resources, Commission on Life Sciences, National Research Council [28]. The permission to carry out animal studies was granted (Approval number: BERC 005-05-19) by the Animal Ethics Committee, College of Pharmacy, Prince Sattam bin Abdulaziz University, AlKharj, Saudi Arabia.

#### 3.10.2. Study Design

Thirty (30) rats were used and divided into five groups (each groups with 6 rats). These group label as “sham control” “colitis control (Acetic Acid; AA group)” “treatment groups; eluxadoline (ELX; mg/kg)” “ELX loaded SLNs (F2; 10 mg/kg)”, and the “positive control group (Prednisolone; 2 mg/kg)”. Group 1 (sham control) and group 2 (AA) were administered with the normal saline only (10 mL/kg), for 6 consecutive days. The animals from groups 3, 4, and 5 were treated with ELX (10 mg/kg), F2 (10 mg/kg), and prednisolone (2 mg/kg), respectively, for 6 consecutive days. All medications were administered orally (PO) once a day for 6 consecutive days and the last dose was administered 2 h before colitis induction.

#### 3.10.3. Induction of Acetic Acid Colitis

Intra-rectal (IR) administration of acetic acid (AA) is a simple technique to induce colitis, which has ulcerative colitis (UC) features in humans, including diarrhea, rectal bleeding, and inflammation. The rats were partially anaesthetized using chloral hydrate (400 mg/kg, IP) after fasting for 16 h.

To induce colitis (in all groups except the sham group), 1.0 mL of AA (5% *v*/*v*, in normal saline) was administered through a polyethylene catheter intra-rectally which was introduced into the rectum such that the tip was 8 cm inside the anus. Then, the rats were held vertically in a head-down position for 60 s to avoid any spillage of the solution. Then, the rectum was washed with 0.5 mL normal saline. Disease index was evaluated after a day of colitis induction, and the rats were then euthanized by dislocation of the cervical vertebrae and colonic biopsies were taken for study purposes. A small part of colon was placed in 10% formalin solution for histopathological examination and the remaining part preserved at −80 °C until the biochemical analysis of oxidative stress parameters (MDA, CAT, and GSH).

#### 3.10.4. Assessment of Disease Activity Index (DAI)

The DAI is the mean of three clinical colitis severity parameters, namely, body weight, stool consistency, and rectal bleeding. These parameters were analyzed before the induction of colitis and until the rats were euthanized in a blinded fashion. The scoring of DAI is described in Table 4 [29,30].

#### 3.10.5. Assessment of Macroscopic Damage

To assess inflammation, the wet weight of the spleen and colon of each rat of the respective group was weighed and its length was measured to calculate the wet weight/length ratios (g/cm) of the colon and spleen samples.

Further, the colon was opened along the mesenteric line and the percentage effected area was estimated, for which 8 cm colon proximal to the anus was evaluated for hyperemia, with or without lesions. Furthermore, to measure ulcer index, the sum of the score of ulcer lesion and ulcer area was measured [31]. The scores of ulcer lesions (0 to 4) were measured [32], which is described as follows: “No changes (0); mucosal erythema (1); mild mucosal edema, mild bleeding or slight erosion (2); moderate edema, bleeding ulcers, or erosion (3); and severe ulceration, erosion, edema and tissue necrosis (4)”. The calculation of ulcer lesion area was done by placing the tissues on graph paper. Each box of graph paper was considered as 1 mm 2 in area and the number of cells was counted and the ulcer area was determined.

Ulcer (UI) = Lesion Score +Area of Ulcer Lesion

#### 3.10.6. Assessment of Lipid Peroxidation Activity

Colon tissues were cut into small pieces and homogenized (10% *w*/*v*) using homogenizer in ice–cold phosphate buffer (0.1 M, pH 7.4). Malondialdehyde (MDA) is an oxidation product of lipids released from the mucosal barrier after oxidative stress. Hence, to check the effect of pure ELX and ELX loaded SLNs (F2) on lipid peroxidation, MDA activity was evaluated in tissue homogenate of the colon of all rats [33].

#### 3.10.7. Assessment of Antioxidant Activity

Antioxidant activity was measured in supernatant of colon homogenate of all animals. Tissue homogenates were centrifuged for 30 min (4 °C) at 12,000 rpm to obtain the PMS (post mitochondrial supernatant). Standard protocols were used to estimate total glutathione [34] and catalase [35].

#### 3.10.8. Microscopic Assessment of UC

After the macroscopic assessment, the intensity of inflammation and ulceration of colon was evaluated by using hematoxylin and eosin (H&E) and goblet cells were assessed by periodic acid Schiff’s (PAS) staining.

Colon tissue samples (3–5 cm), proximal to the anus were excised and immersed immediately in the appropriate amount of neutral buffered formalin and stored for 24 h. The samples were operated in an automatic tissue operating machine (ASP300s, Leica Biosystems, IL, USA) and then fixed in wax, and a 5µm thick section was cut using rotary microtome (SHUR/Cut 4500, TBS, Burlington, NC, USA). Two sections of each sample were selected, one was stained by H&E technique and the other was stained by PAS method for neutral mucin, using standard procedures as mentioned in previous studies.

Histopathological changes in tissues were evaluated by an expert blind to group using an Olympus BX 52 microscope. The photographs of tissues section were taken by a camera (Olympus DP21) attached to the microscope and photographs were labeled using Microsoft PowerPoint 2010 software.

### 3.11. Statistical Analysis

Results were expressed as the mean ± standard error of the mean (SEM). Statistical variations of different treatment groups were analyzed according to one-way analysis of variance (ANOVA) followed by post hoc Tukey’s test. *p* < 0.05 was considered statistically significant. Statistical analysis was performed using the GraphPad Prism program (version 4) (GraphPad Software, San Diego, CA, USA).

## 4. Conclusions

This study shows that ELX loaded SLNs were successfully prepared and evaluated using three different lipids. The stearic acid based SLNs (F2) was found to be optimum, with suitable size, PDI, zeta potential, and drug entrapment to enhance the efficacy of ELX in terms of drug release and stability, which was further confirmed by significant protective effects against acetic acid induced UC. The prepared SLNs could limits the release in the stomach and small intestine and targets the delivery of ELX in the colon.

## Figures and Tables

**Table 1:** Particle characterization of the solid lipid nanoparticles (SLNs).

SLNs	SIZE ± SD (nm)	PDI	ZP ± SD (mV)	%EE ± SD	%DL ± SD
F1	394.3 ± 8.4	0.226 ± 0.03	26.8 ± 5.41	73.0 ± 3.2	5.13 ± 1.2
F2	266.0 ± 6.4	0.217 ± 0.04	31.2 ± 5.19	65.0 ± 4.8	4.60 ± 0.8
F3	1570.5 ± 14.2	0.882 ± 0.06	25.7 ± 6.11	54.1 ± 2.6	3.82 ± 0.7

**Table 2:** Stability data of optimized ELX loaded SLNs (F2).

Months	Conditions	Particle Size (nm ± SD)	PDI (± SD)	ZP (mV) (mV ± SD)	Entrapment Efficiency (% ± SD)	Release (% ± SD)
0	-	266 ± 6.4	0.217 ± 0.04	31.2 ± 5.1	65.0 ± 4.8	95.7 ± 4.7
1	30 ± 2 °C/65 ± 5% RH	268 ± 4.5	0.243 ± 0.02	30.3 ± 6.3	67.2 ± 2.7	96.5 ± 3.1
2		272 ± 7.4	0.249 ± 0.06	27.2 ± 5.8	63.6 ± 3.9	94.6 ± 4.4
3		276 ± 8.5	0.212 ± 0.04	30.5 ± 4.2	62.4 ± 8.3	91.4 ± 7.3
1	40 ± 2 °C/75 ± 5% RH	267 ± 2.5	0.223 ± 0.07	33.6 ± 9.4	65.2 ± 4.8	93.6 ± 6.4
2		273 ± 6.8	0.279 ± 0.09	29.2 ± 6.3	63.6 ± 2.6	93.2 ± 7.3
3		276 ± 7.5	0.272 ± 0.08	25.7 ± 6.7	61.5 ± 5.9	90.8 ± 6.2

**Table 3:** Composition of ELX loaded SLNs.

SLNs	ELX (mg)	Lipids (400 mg)	Soyalecithin (%w/v)
F1	40	Glyceryl monostearate	0.5
F2	40	Stearic acid	0.5
F3	40	Glyceryl trioctanoate	0.5

**Table 4:** Scoring of disease activity index.

Weight Loss (%)	Stool Consistency	Occult/Gross Bleeding	Score
Normal	Normal	Normal	0
1–5	Soft but still formed	-	1
5–10	Loose stools	Hemo-occult positive	2
10–20	Diarrhea	-	3
>20	-	Gross bleeding	4
